# Supplementary figures and images for: Axon initial segment geometry in relation to motoneuron excitability
Source: PLoS One. 2021 Nov 19;16(11):e0259918. doi: 10.1371/journal.pone.0259918 (PMC8604372; doi:10.1371/journal.pone.0259918)

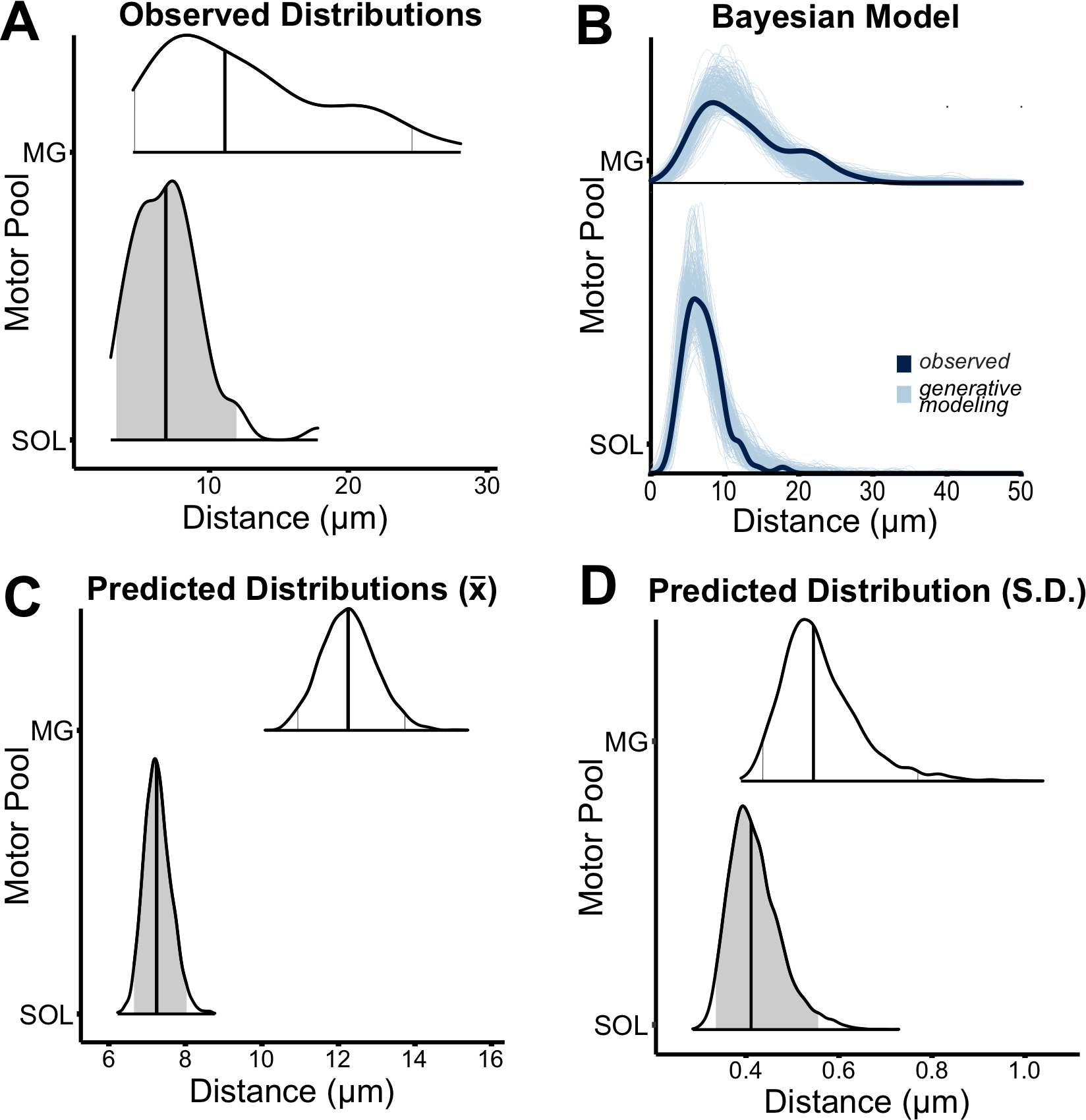

Supplement: S1 Fig — A) Distribution of the observed AIS distance between the MG (n = 65) and SOL (n = 82) motoneurons. Black vertical line inside the plot represents the median value. MG: avg. 12.50 ± 5.95 (s.d.), SOL: 7.11 ± 2.65 (95% HDI—SOL: 6.58–7.84, MG: 11.0–14.4). B) Bayesian posterior predictive modeling distributions for AISd in MG and SOL motor pools conditioned on the observed data in A). C) Average and D) standard deviation derived from shifted log-normal regression model (see Methods). (TIF) [file pone.0259918.s001.tif]

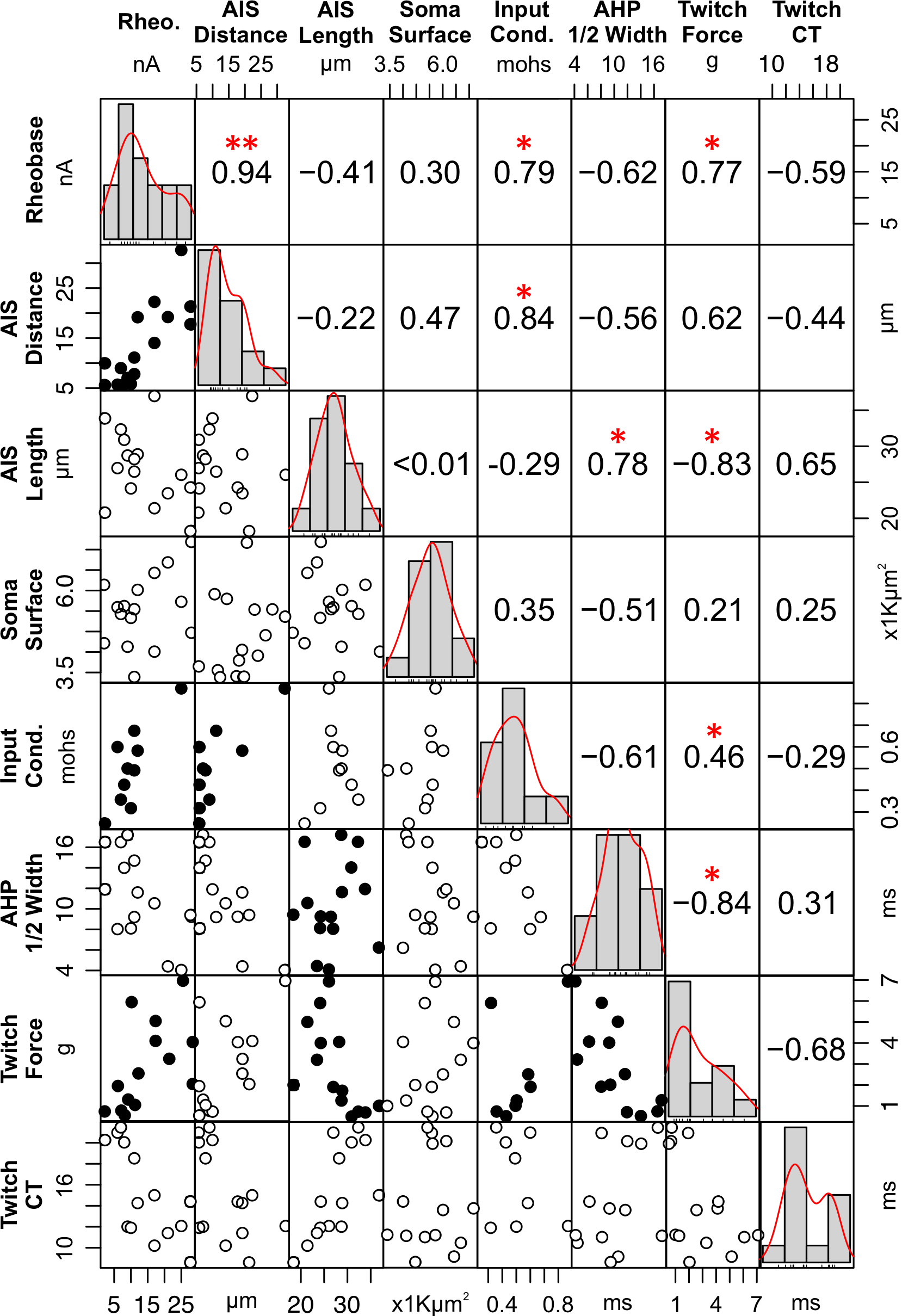

Supplement: S2 Fig — Upper left to the bottom right diagonal: Histogram plots showing distribution of data from motoneurons. Fitted line to the distribution of data is in red. Left of histogram: Scatter plots for all comparisons. Each dot represents a single data point. The open white circles represent comparisons that do not reach significance. All black circles represent significant correlations. Right of the histograms: Each value listed in the matrix is an r value computed from a Pearson correlation. The number of red asterisks refer to significance level corrected for multiple comparisons (*p<0.05, **p<0.01, ***p<0.001). (TIF) [file pone.0259918.s002.tif]
