# Supplementary material for: Axon initial segment geometry in relation to motoneuron excitability
Source: PLoS One. 2021 Nov 19;16(11):e0259918. doi: 10.1371/journal.pone.0259918 (PMC8604372; doi:10.1371/journal.pone.0259918)
Supplement: Attachment — Submitted filename: Response to Reviewers .pdf [file pone.0259918.s003.pdf]

Georgia Institute of Technology

School of Biological Sciences
Atlanta, Georgia 30332-2230 U.S.A.
PHONE 404.894.3700
FAX 404.894.0519

October 22nd, 2021

Dear Reviewers,

We would first like to thank both reviewers for their careful consideration and detailed assessment of our manuscript entitled, "Axon initial segment geometry in relation to motoneuron recruitability." We found the comments to be constructive, and by addressing each of them in the revised edition, we have improved the overall quality and presentation of our work. Below we provide responses indicating how we edited the paper to address each individual point raised by the academic editor and both reviewers below:

Reviewer 1:

- We have elaborated on possible interpretations of our data including variability in spatial distribution and density of voltage-gated channels at the AIS within the discussion section. We also included mechanistic explanations and have provided both primary and review articles to support our claims. We agree with the reviewer that this will prove beneficial to the broad audience of PLOS One.

Reviewer 2:

- In reference to line 265: We thank you for catching this. This was an oversight on our part. Our intent is was to show two additional data points on the graph to display two filled motoneurons that had axons originating from the dendrites though the values of these two cells were not actually included in the statistical analysis. However, they exceeded the limits of the plot. The sentence has been fixed to reflect the current figure.
- In reference to line 364: This is an excellent point and we have elaborated on this in the discussion however threshold was not included in our final analysis due to experimental variables that impacted our ability to collect a complete and comprehensive dataset. A common occurrence while using 10% Neurobiotin is an increase in electrode resistivity and rectification during current injection which was necessary to unblock some action potentials. This made it challenging to measure voltage-threshold accurately and rigorously in our sample.

Again, we thank both reviewers and the editorial staff for their time and attention to our work.

Timothy Cope

Timothy Cope
Professor, School of Biological Sciences and Department of Biomedical Engineering
Georgia Institute of Technology
404-385-4293
tim.cope@gatech.edu
